# Can polycythaemia vera disease be predicted from haematologic parameters? A machine learning-based study

**DOI:** 10.1136/jcp-2025-210087

**Published:** 2025-07-23

**Authors:** Murat Haskul, Emin Kaya, Ahmet Kurtoğlu

**Affiliations:** 1 Department of Medical Oncology, Inonu University, Malatya, Turkey; 2 Department of Haematology, Inonu University, Malatya, Turkey; 3 Department of Coaching Education, Bandirma Onyedi Eylül University, Balikesir, Turkey

**Keywords:** Polycythemia Vera, Machine Learning, Blood Platelets, Hematology

## Abstract

**Aims:**

The aim of this research is to diagnose polycythaemia vera (PV) disease using different machine learning (ML) algorithms with complete blood count (CBC) parameters before further investigations such as Janus kinase 2 (*JAK2*), erythropoietin (EPO) and bone marrow biopsy (BMB).

**Methods:**

The study included 1484 patients who presented to the adult haematology clinic with elevated haemoglobin. Participants were retrospectively screened for *JAK2*, EPO and BMB results, and patients were categorised as PV group (n=82) and non-PV (other) (n=1402). First, the synthetic minority oversampling technique (SMOTE) method was used to avoid data imbalance. Then, classification predictions were made using Random Forest, Support Vector Machine Technique, Extreme Gradient Boosting (XGBoost) and K-Nearest Neighbours algorithms according to the participants’ CBC parameters of white cell count (WBC), haematocrit (HCT), haemoglobin (HGB) and platelet (PLT).

**Results:**

The XGBoost algorithm was found to be the most effective ML algorithm in predicting the model (area under the curve=0.99, accuracy=0.94, F1-Score=0.94). In addition, the most effective parameter in the prediction of the model was PLT with 42.4%. As a result of the t-test, there was a highly significant difference between the WBC, PLT, HGB, HCT, EPO, *JAK2* and bone marrow density results of PV and other groups (p<0.001).

**Conclusion:**

ML algorithms can diagnose PV with CBC parameters with high accuracy, thus emphasising the potential to reduce the dependence on costly diagnostic methods such as *JAK2*, EPO and BMB.

**WHAT IS ALREADY KNOWN ON THIS TOPIC**

- In the literature, there are standardised major and minor criteria in cases with erythrocytosis. Some of these criteria are very costly and stressful tests on the patient. We thought that machine learning (ML) and artificial intelligence algorithms, which have been frequently used in recent years to determine the relationship between such complex data sets, would provide important findings in the diagnosis of polycythaemia vera (PV).

**WHAT THIS STUDY ADDS**

- In our model built using different ML algorithms, PV prediction was performed with high accuracy in routine complete blood count analysis without the need for costly and troublesome tests.

**HOW THIS STUDY MIGHT AFFECT RESEARCH, PRACTICE OR POLICY**

- The results of this study show that PV can be diagnosed with less energy, time and cost for both clinicians and patients with erythrocytosis by giving important results to experts before further investigations such as JAK2 and bone marrow biopsy.

## Introduction

 Increased haemoglobin and/or haematocrit in peripheral blood is called erythrocytosis.^1^ Erythrocytosis is usually detected incidentally during routine investigations. Erythrocytosis caused by a decrease in plasma amount due to dehydration is called relative erythrocytosis.^2^ In absolute erythrocytosis, there is an increase in the amount of erythrocytes.^3^ Absolute erythrocytosis is defined as haemoglobin (HGB)>16.5 g/dL (10.3 mmol/L) for men and >16.0 g/dL (10. 0 mmol/L) for women or haematocrit (HCT)>49% for men and HCT>48% for women, or an increase in the number of erythrocytes by 25% or more compared with the average haemoglobin level expected for the adult gender and age group.^4^

Absolute erythrocytosis is divided into three subclasses: primary erythrocytosis caused by an intrinsic defect in the precursor cells of the haematopoietic series, secondary erythrocytosis caused by extrinsic stimuli such as erythropoietin (EPO) and idiopathic erythrocytosis in which no aetiological cause is identified.^5^ The prototype of primary erythrocytosis is PV. PV is defined as an acquired stem cell disease characterised by uncontrolled clonal production of precursor cells with normal morphology of erythrocytes, platelets (PLT) and leukocytes in the absence of any stimulus and extramedullary haematopoiesis. Although the aetiology of the disease has not been fully elucidated, the incidence is 5–17/million in the USA and Europe. PV is more frequently seen in people older than 60 years of age, but is extremely rare in people younger than 40 years of age.^6^ Although the incidence is similar in men and women, it is slightly higher in men.^7^ At the time of diagnosis, splenomegaly is found in 70% of patients and hepatomegaly in 30%. Therefore, PV patients usually have mild to moderate leucocytosis and thrombocytosis.^8^ Leucocytosis is found in 60% of patients with PV, and thrombocytosis in half of the patients.^9^ Serum EPO level is frequently low in PV. The V617F point mutation in exon 14 of *JAK2* is present in 95% of patients with PV.^10 11^ Currently, the diagnostic criteria of the updated International Consensus Classification (ICC) are used for PV. The diagnostic criteria for PV require either all three major criteria or the first two major criteria and minor criteria to be met. These major criteria are as follows: HGB>16.5 g/dL in men, >16.0 g/dL in women; HCT>49% in men, >48% in women, bone marrow biopsy showing hypercellularity for age with trilineage enlargement (panmyelosis) including marked erythroid, granulocytic and megakaryocytic proliferation with pleomorphic, mature megakaryocytes (size differences) and presence of *JAK2 V617F* or *JAK2* exon 12 mutation. Minor criteria are subnormal serum EPO level (normal range of EPO in adults: 4.1–19.5 mU/mL). Major criterion 2 (bone marrow biopsy) may not be necessary in cases with persistent absolute erythrocytosis (persistent absolute erythrocytosis is defined as HGB levels higher than 18.5 g/dL in men or 16.5 g/dL in women or HCT higher than 55.5% in men and 49.5% in women). Accordingly, three major criteria or the first two major criteria plus minor criteria must be present for the diagnosis of PV.^11^

As in the diagnosis of PV, major and minor diagnostic tests are used in the diagnosis of many diseases, and these tests take time to be completed and analysed by a specialist. In addition, these high-cost tests have negative effects on healthcare costs and the patient. For this reason, machine learning (ML) and artificial intelligence (AI) algorithms have been frequently used in disease diagnosis in recent years and make high-accuracy predictions with different algorithms among existing diseases with simpler analyses.^12^ In their study, Mujumdar and Vaidehi predicted diabetes from external factors such as age, BMI, glucose, insulin, etc, and reached an accuracy of 96% with the Logistic Regression model.^13^ In the study by Uddin *et al*, in which the results of research using many ML algorithms were compared, the results of different algorithms were compared, and the results of the algorithms used were shared.^14^ As can be seen from the results of these studies, algorithms such as Random Forest (RF), Support Vector Machines (SVM), Extreme Gradient Boosting (XGBoost) and K-Nearest Neighbour (KNN) are widely used to analyse large data sets, and the effectiveness of the models used is evaluated with metrics such as accuracy, recall and Receiver Operating Characteristic (ROC) curve.^15^ Analysis methods such as Shapley Additive explanations (SHAP) plots also provide us with the opportunity to see which feature has an impact on the model and how much.^16^ These advanced analysis methods have the potential to not only diagnose disease but also to reduce the reliance on costly and invasive diagnostic methods, leading to significant improvements in medical practice.

In this context, this study aims to predict PV disease, which requires advanced tests such as *JAK2*, bone marrow biopsy and EPO during the clinical evaluation process, by using different ML algorithms with routine haemogram parameters without the need for these advanced tests. In this context, the hypothesis of our study was determined as ‘PV disease is predicted with high accuracy using haemogram parameters with ML algorithms’.

## Methods

### Patient and public involvement

Patients with erythrocytosis detected in the Adult Haematology Outpatient Clinic of the Faculty of Medicine between January 2010 and August 2021 were included in the study by scanning through the electronic record system. This study was designed retrospectively using data from patients between the specified years. Therefore, the patients were not directly involved throughout the study. For this, the hospital registration system was used, and necessary permissions were obtained from the hospital administration. Then, all data were retrospectively reviewed, and the cases that met the inclusion and exclusion criteria were included in our study. In this study, direct consent was not obtained from the patients. The identity information of the hospital participants was kept confidential, and their data were analysed. All data were anonymised before being analysed, and only necessary information was used. In this context, the files of 1484 patients with erythrocytosis were scanned through the hospital automation system by analysing retrospective electronic records and laboratory values. HGB, HCT, WBC, PLT, presence or absence of *JAK2* mutation, bone marrow investigations, EPO level and PV diagnosis at the time of diagnosis were analysed, and the available data were recorded. Patients aged 18–99 years with erythrocytosis (HGB>16.5 g/dL in males and HGB>16.0 g/dL in females) in the complete blood count were included in the study. Participants under 18 years of age, those with inaccurate data, individuals with high haemoglobin levels associated with haematological malignancies such as leukaemia, myelodysplastic syndromes or other systemic diseases, individuals with pregnancy, individuals with a history of PV-related treatment such as *JAK2* inhibitors, EPO therapy or chemotherapy and participants with insufficient clinical follow-up information were excluded.

This research was approved by the Health Sciences Non-Interventional Clinical Research Ethics Committee on 15 November 2022, 18th session, and decision number 2022/3787. The study was conducted in accordance with the principles set out in the Declaration of Helsinki.

### Data collection tools

#### Janus kinase 2 (JAK2) test

The real-time PCR method was used for the detection of the *JAK2 (V617F*) mutation for the diagnosis of PV. *JAK2* exon 12 mutation analysis was performed in addition to the *JAK2 V617F* mutation. In this way, a second analysis was performed in the diagnosis of PV for those who were not positive for the *JAK2 V617F* mutation (n=254). However, the *JAK2* exon 12 mutation test performed on all of these patients was negative. In this process, a peripheral blood sample was carefully taken from the patient and prepared for mutation analysis. DNA was first isolated from a blood sample. This process was performed by standard phenol-chloroform extraction. The quality and concentration of the obtained DNA sample were confirmed by spectrophotometric methods. Commercial kits containing specific primers and probes for *JAK2 V617F* and exon 12 mutations were used. Real-time PCR analysis targeted the V617F mutation in the *JAK2* gene using specific primers and probes. The PCR mixture was prepared with isolated DNA, a specific primer/probe set and a Taq DNA polymerase enzyme. This reaction was performed in a thermocycle device. A fluorescent signal was generated during amplification and the presence of the mutation was analysed. During the analysis process, the signal exceeding the Ct value indicated that the mutation was positive. These data were analysed in the software of the real-time PCR device, and the final result was obtained.^17^

### Bone marrow biopsy

Another method frequently used in the diagnosis of PV disease is bone marrow biopsy. This analysis is an important method to evaluate the haematopoietic activity of the patient and to confirm myeloproliferative diseases. In this procedure, bone marrow samples were obtained from the posterior iliac crest of the patients under local anaesthesia. A fine biopsy needle was used during the procedure and both bone marrow aspiration and bone marrow biopsy were obtained. During aspiration, a liquid bone marrow sample was obtained and examined under a microscope using the Wright-Giemsa staining method. A bone marrow biopsy was performed by providing a solid tissue sample and histopathological evaluation of the cellular structure. The biopsy material obtained was fixed and then processed into paraffin blocks. Thin sections taken from these blocks were examined using reticulin staining and H&E staining. During the examination, proliferation of erythroid, myeloid and megakaryocyte cell lines was evaluated. Hyperplasia and megakaryocyte clusters, which are typical of PV patients, were observed.^18^

### Complete blood count (CBC) analyses

Complete blood count (CBC) of the participants was determined with a venous blood sample obtained from the patient. The sample was collected in a tube containing EDTA anticoagulant and run on an analyser. Haemoglobin (HGB), HCT, white blood cell (WBC) and PLT values were obtained in CBC analysis.

### Erythropoietin (EPO) analysis

Analysis of EPO level is an important test in the diagnosis of PV, and a low EPO value indicates the myeloproliferative nature of the disease.^19^ For this analysis, a venous blood sample was first obtained from the patient. After the sample was centrifuged, the serum portion was separated and prepared for the test. The analyses were performed by chemiluminescence techniques. In this method, EPO in the serum part of the blood reacts with specific antibodies to produce a signal, and the intensity of this signal determines the EPO level. Generally, the EPO level in PV patients is due to the *JAK2* mutation, causing an increase in erythropoiesis through independent EPO signalling pathways.^20^

### Machine learning approach

ML is a field of AI that extracts meaningful patterns from big data and patterns using artificial neural networks and facilitates prediction or decision-making processes.^21 22^ In the study, ML methods were used to predict PV diagnoses from the haemogram parameters of PV-positive and negative cases who came to the hospital adult haematology outpatient clinic with elevated haemoglobin. In this context, four different ML algorithms, RF, SVM, XGBoost and KNN algorithms were used. In testing the ML algorithms, data preprocessing was performed first. Accordingly, after loading the data into the Python 3 programme, healthy individuals were coded as 0 (zero) and patients with PV were coded as 1 (one) for disease prediction. The haematological parameters to be analysed were selected (HGB, HCT, WBC, and PLT). Missing data in the data set were filled with the mean of the relevant parameter, as missing data would negatively affect the analysis process. The PV estimated by ML was supervised by a blinded reviewer.

### SMOTE (Synthetic Minority Oversampling Technique)

In our study, there was a large imbalance between the groups with (n=82) and without (n=1402) PV diagnosis. Therefore, the SMOTE method was preferred to overcome the data imbalance.^23^ For the SMOTE method, the *imblearn* library was used in the Python programme. This technique is a valid and reliable ML method used to improve the performance of the model, especially in classification problems with severe imbalances between classes.^24^ The SMOTE method calculates KNN for each data point from the minority class.^25^ The neighbourhood information is used as a basis for generating new data samples. The following formula is used when generating synthetic data:


(1) xnew=xi+r (xneighbor−xi)

**Figure 1:**
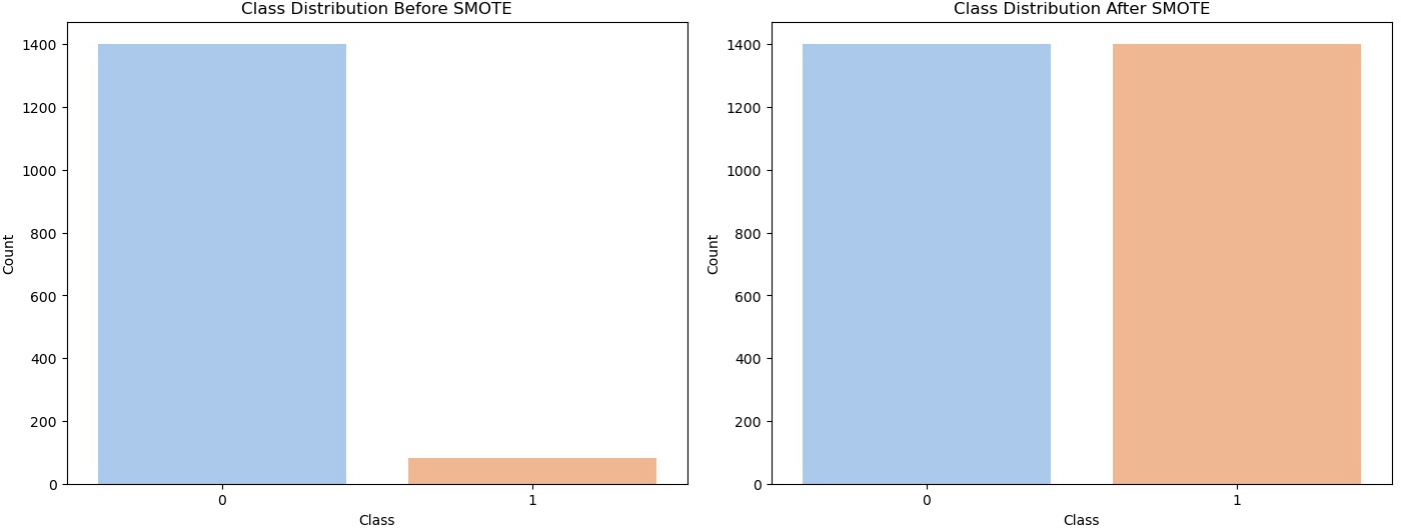
Synthetic minority oversampling technique. SMOTE, synthetic minority oversampling technique.

The distribution of data before and after SMOTE is given in figure 1.

After the imbalance in the data set was removed by the SMOTE method, the data set was split into 80% training data and 20% test data. The stratified split method was used to ensure a balanced distribution between classes. In this way, we tried to create an ideal structure to measure the performance of the model in recognising both PV and non-PV patients. Furthermore, basic principles such as randomness control and prioritisation of data temporalisation were also taken into account.

### Machine learning algorithms

#### Random forest (RF)

The RF algorithm technique is an ensemble learning method, which is a technique often used in ML analysis and is often used in classification and regression analysis. This algorithm provides better prediction performance by creating multiple decision trees (DT) and combining the decisions of these trees. This model is notable for its ability to work with non-linear data structures and reduce overfitting.^26^

#### Support Vector Machine (SVM) Technique

SVM is a powerful supervised learning algorithm used in classification and regression problems. In basic principle, SVM works by creating an optimal hyper-regularisation that separates data points into different classes. SVM creates a plane that maximises the margin between classes. Margin is the distance between the hyperplane and the closest data points (support vectors) of the classes. If the data set is not linearly decomposed, SVM uses kernel functions to transform the data set into a higher dimensional space. In this study, the radial basis function kernel is preferred for nonlinear data:


(2) K(xi,xj)=exp(−gamma∣∣xi−xj∣∣2)

#### Extreme Gradient Boosting (XGBoost)

The XGBoost ML method is based on a gradient boosting algorithm, optimised especially for speed and performance. It is used for both classification and regression models. This method gives significant results in terms of accuracy and generalisability, especially for large data sets.^27^

#### K-Nearest Neighbours (KNN)

KNN is a supervised ML algorithm. It is used for both classification and regression problems. KNN is based on the proximity to one of the data points in the feature space. Accordingly, it looks at the classes of the KNN of a data point when determining the value of the target variable. For classification in this study, voting was done based on the classes of k neighbours and the class with the highest number of votes was predicted.^28^


(3) d (j, l)=|xj1−xl1|+|xj2−xl2|+|xj3−xl3|

In this research, we used the Confusion Matrix to visually describe the evaluation metrics for the number of labels of correct and incorrect prediction examples for the four prediction models.^29^ The effectiveness and reasonableness of the four models for predicting PV were evaluated. After the evaluation, the comprehensive evaluation indices (accuracy, recall, precision, F1-Score and area under the curve (AUC)) of the most accurate values were analysed. The formulas for these data are as follows:


(4) $$Accuracy=\frac{TP+TN}{TP+TN+FP+FN}$$

(5) $$Recall=\frac{TP}{TP+TN}$$

(6) $$Precision=\frac{TP}{TP+FN}$$

(7) $$F1\;Score\;=2\times \frac{Precision\times Recall}{Precision+Recall}$$

For AUC value:


(8) $$True\;Positive\;Rate\;\left(TPR\right)=\frac{TP}{TP+FN}$$

(9) $$False\;Positive\;Rate\;\left(TPR\right)=\frac{FP}{FP+TN}$$

(10) $$AUC=\int_{0}^{1}TPR\times d\left(FPR\right)$$

### Statistical analysis

In the study, statistics other than ML analyses were performed with the SPSS package programme V.25 (IBM, Chicago, USA). First, normality analyses of all the data obtained were performed by Kolmogorov-Smirnov test, and it was determined that the data were normally distributed. In addition, Levene’s test was applied for homogeneity of variances. The χ^2^ test was used for statistical analyses of the categorical variables of *JAK2* positive and negative, bone biopsy results as normal, compatible with MPN and other categorical variables, presence and absence of splenomegaly, in patients with PV and other patients. An independent sample t-test was used to analyse the differences in HGB, HCT, WBC, PLT and EPO between participants with PV diagnosis and other patients. The level of significance was set as 0.05.

## Results

Table 1 shows the results of the Independent Sample T Test of haemogram parameters and EPO results of the participants. There was a statistically significant difference between these two groups (p<0.05). When haemoglobin, haematocrit, WBC and PLT levels of both groups were compared, mean values were found to be higher in patients with PV compared with patients in the other group in all parameters, and a statistically significant difference was observed (p<0.05). When the patients included in the study in whom EPO was studied were compared in terms of both groups, the median value was found to be 1.77 (U/L) in patients diagnosed with PV and 9.87 (U/L) in the others. EPO was found to be lower in patients diagnosed with PV, and a statistically significant difference was found when compared with the others (p<0.05).

**Table 1:** Comparison of CBC parameters and EPO levels between groups

	PV			Other			t	P value
	N	$\bar{X}$	SD	N	$\bar{X}$	SD		
HGB (g/dL)	82	18.14	1235	1402	17.69	0.80	85.05	0.002*
HCT (%)	82	56.23	5144	1402	51.60	3.30	84.94	<0001*
WBC (10^3^ /uL)	82	11.90	3394	1402	8.71	2.87	87.90	<0001*
PLT (10^3^ /uL)	82	517.87	292.07	1402	238.01	83.43	81.78	<0001*
EPO (U/L)	39	1.77	1013	755	9.87	13.32	−3.79	<0001*

* P<0.05 significant difference.

CBC, complete blood count; EPO, erythropoietin; HCT, haematocrit; HGB, haemoglobin; PLT, platelet; PV, polycythaemia vera; WBC, white blood cells.

*JAK2* and bone marrow biopsy results of the groups are compared in table 2. In our study, *JAK2* positivity was found to be 97.5% in patients diagnosed with PV. A statistically significant difference was found between both groups (p<0.05). When these two groups were compared according to the results of bone marrow biopsy, 29 (76.3%) of 82 patients diagnosed with PV had bone marrow biopsy results compatible with myeloproliferative disease, while this number was 6 (8.8%) in 1402 patients in the other group. When both groups were compared, a statistically significant difference was found (p<0.05). In addition, EPO value was found to be below the limit (EPO<4.1) in 5.03% of the participants without PV diagnosis.

**Table 2:** Comparison of the groups with *JAK2* and bone marrow biopsy results

Tests	Groups	PV N (%)	Other N (%)	Total N (%)	X^2^	SD	P value
*JAK2*	Negative	2 (2.5)	1032 (97.8)	1034 (91.1)	833 419	1	<0001*
	Positive	78 (97.5)	23 (2.2)	101 (8.9)			
Bone marrow biopsy	Normal	0 (0)	14 (20.6)	14 (13.2)	51 427	2	<0001*
	Compatible with MPN	29 (76.3)	6 (8.8)	35 (33)			
	Other	9 (23.7)	48 (70.6)	57 (53.8)			

*JAK2*, Janus kinase 2; PV, polycythaemia vera.

**Figure 2:**
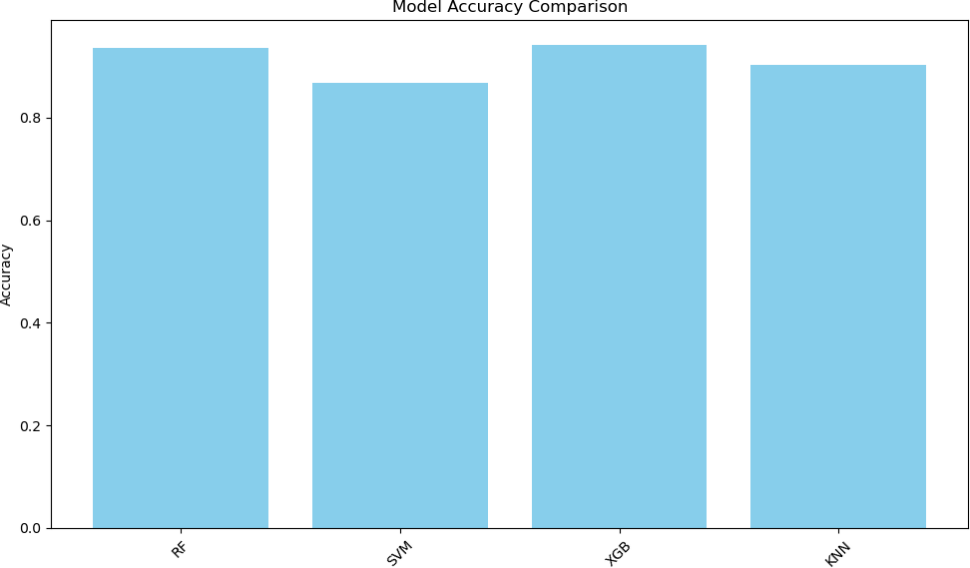
Comparison of ML algorithms. ML, maximum likelihood; XGBoost, Extreme Gradient Boosting.

Statistical analyses confirmed the diagnosis of PV disease as a result of *JAK2* and bone marrow biopsy. As a result, results related to ML algorithms will be presented. Table 3 shows the findings obtained from the execution of the ML algorithms. Accordingly, the findings from the XGBoost algorithm had a higher accuracy rate (figure 2).

**Table 3:** Comparison of the results of algorithms used in PV estimation

Model	Accuracy	Precision	Recall	F1-Score
RF	0.93	0.92	0.95	0.93
SVM	0.86	0.81	0.94	0.87
XGBoost	0.94	0.93	0.95	0.94
KNN	0.90	0.90	0.90	0.90

KNN, K-Nearest Neighbours; PV, polycythaemia vera; RF, Random Forest; SVM, Support Vector Machine; XGBoost, Extreme Gradient Boosting.

**Figure 3:**
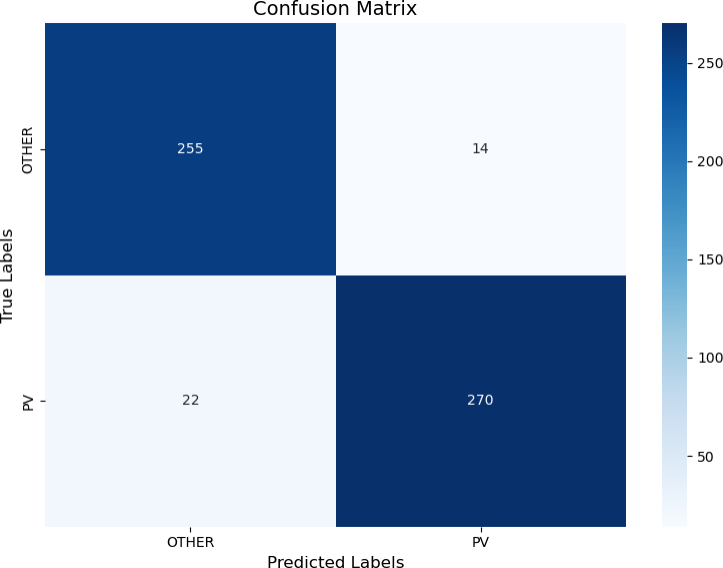
Confusion matrix for classification model performance. PV, polycythaemia vera.

Figure 3 shows the confusion matrix to evaluate the performance of the XGBoost algorithm for participants. Accordingly, the model correctly classified 255 of the non-patients and 270 of the PV patients. It classified 14 samples as other despite being PV and 22 samples as PV despite being in the other patient group.

**Figure 4:**
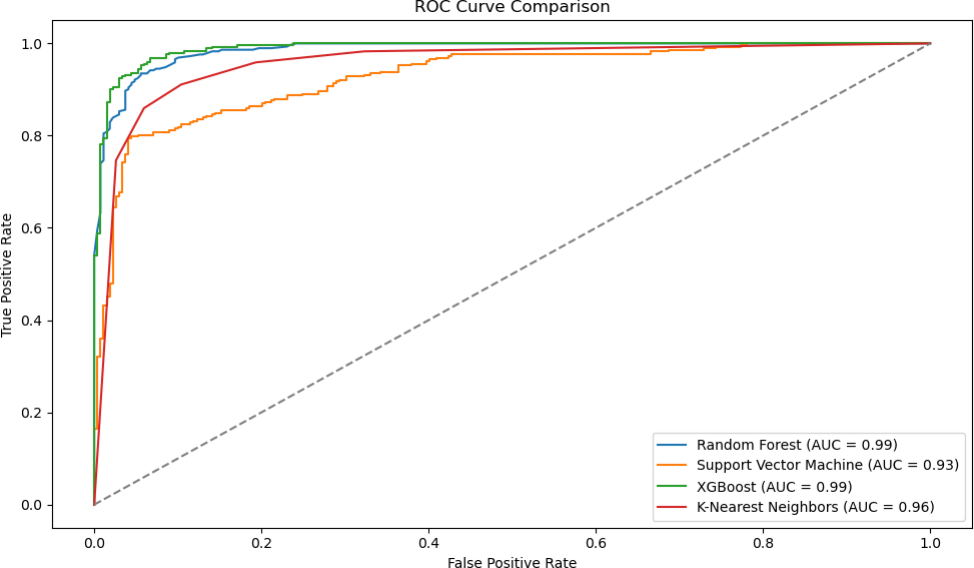
ROC curve of ML algorithms. ML, maximum likelihood; ROC, receiver operating characteristic.

Figure 4 shows the ROC curve plot and AUC values for each model to measure the classification performance of different ML algorithms. The ROC curve visualises the model’s ability to distinguish between positive and negative classes, while the AUC value was used to express this performance numerically. Accordingly, the ROC curve value for ML analysis with RF and XGBoost algorithms was found to be 0.99, indicating a near-perfect classification accuracy. KNN (AUC=0.96) and SVM (AUC=0.93) classification also had a moderate accuracy.

**Figure 5:**
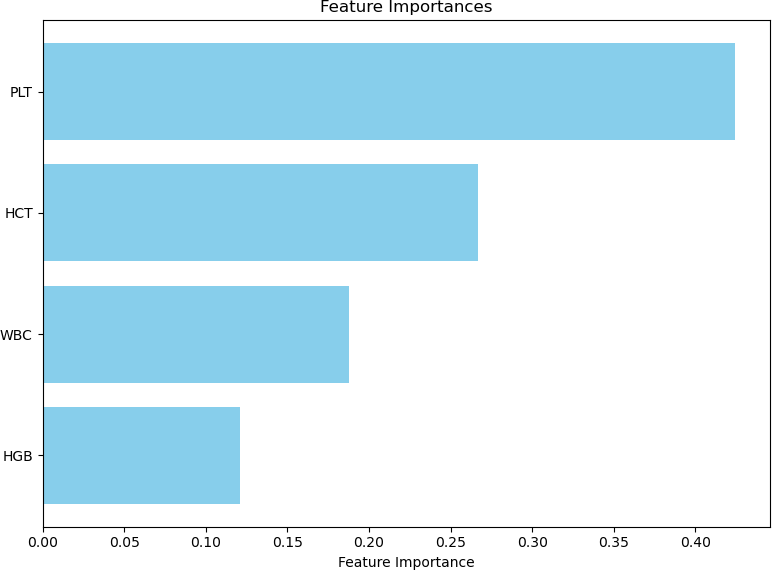
Examination of the effects of classification parameters on the model. HCT, haematocrit; HGB, haemoglobin; PLT, platelet; WBC, white blood cell.

Figure 5 shows the effects of the classification parameters (PLT, HCT, WBC and HGB) obtained with the XGBoost algorithm on the algorithm. Accordingly, the most influential parameter on the model is PLT (42.4%), followed by HCT (26.7%), then WBC (18.7%), and the least influential parameter is HGB (12.0%).

## Discussion

In our research, ML algorithms, which have been the subject of current research in medical sciences as well as in many different disciplines in recent years, were used in the diagnosis of PV. In our research, algorithms such as RF, SVM, XGBoost and KNN were used in PV diagnosis and the highest accuracy rate was obtained with the XGBoost algorithm. In addition, CBC parameters were utilised in the prediction of PV disease, and PLT, HCT, WBC and HGB parameters were found to have high accuracy values in predicting the model. PLT had the highest effect value in predicting the model. Our model predicted both PV and non-PV groups with high accuracy, and our hypothesis ‘PV disease is predicted with high accuracy using haemogram parameters with ML algorithms’ was confirmed. To the best of our knowledge, this is the first study in which ML algorithms were used to diagnose PV using CBC parameters.

Accurate diagnosis of PV, which is also defined as a clonal neoplasia within myeloproliferative diseases and is an important cause of mortality and morbidity due to its complications, is of great importance. For this reason, many diagnostic criteria have been developed in the historical process, and the latest ICC diagnostic criteria are currently used. In every period, including the current diagnostic criteria, some issues, such as inadequacy of the diagnostic criteria or unnecessary further investigations in a large group, have been criticised. For example, Iurlo *et al* stated that in addition to diagnostic criteria such as *JAK2* and bone marrow biopsy, EPO is frequently used as a minor criterion in the diagnosis of PV.^30^ Davila-Gonzalez *et al* argued that cytogenetic and molecular studies such as *JAK2* mutation status are the most useful investigations in the identification of PV cases, but isolated EPO use is not a good diagnostic approach.^31^ Silver and Abu-Zeinah concluded that bone marrow biopsy is important for diagnosis and basic histomorphology in PV and that both HCT and WBC counts should be controlled by phlebotomy and cytoreductive agents. One of the most important conclusions of this study is that the authors emphasised the need for short-term biomarkers that predict long-term outcomes.^32^ Barrios-Ruiz *et al* investigated the potential effects of diagnostic standard codes in differentiating PV from secondary erythrocytosis and concluded that only standardised code systems, such as the International Classification of Diseases, cause some problems in classifying PV disease.^33^ This situation necessitates the use of systems such as ML and AI algorithms, which allow in-depth examination of the effects of many different physiological factors on each other, in the diagnosis of disease, and there have been many studies on this subject in recent years.^12^

ML methods are nowadays becoming more and more common in the diagnosis and prediction of faults. ML methods offer significant advantages in terms of high accuracy rates on multidimensional and complex data. In addition, one of the most important advantages of ML and AI methods is that they facilitate the understanding of these physiological processes by recognising different artificial networks among very complex physiological outputs, especially in the field of medical sciences. In this context, when research on ML and disease prediction is analysed, it is seen that many different methods have been tried. For example, Shah *et al* used naive Bayes, DT, KNN and RF algorithms in the prediction of heart diseases, and the highest accuracy rate was achieved with the KNN algorithm.^34^ In the ML application for the prediction of this disease in individuals with diabetes-induced heart disease, DT reached a very high accuracy with an accuracy rate of 90% and predicted heart diseases at a high level in patients with diabetes.^35^ As can be seen, in different studies, the highest accuracy rate has been achieved with different algorithms. This is due to the different characteristics of the data set. In our research, we achieved the highest accuracy rate with the XGBoost algorithm, which is known to perform quickly and efficiently on large data sets. Its ability to work with missing data is also better than other algorithms.^36^ It also makes more precise and effective decisions when using tree structure-based optimisation.^37^

One of the most important outputs of this study is the relationship between PLT, HCT, WBC and HGB parameters and overall prediction performance. According to the data obtained, the PLT model had the highest prediction value. EPO levels are below normal in PV patients but may be normal in up to one-third of patients. As mutation assays are costly and require access to specialised laboratories, in a recent study, neutrophil/lymphocyte ratio or PLT/lymphocyte ratio is rapidly available biomarkers to identify PV patients at increased risk of thrombosis and measurement.^38^ In this study, PLT/neutrophil ratio predicted PV disease with a rate of 93.6%, while this rate was 99% in our study and we obtained a higher result. Considering the sample numbers of the study conducted by Krečak *et al* (PV=103, non-PV=104), the accuracy rate obtained in our study is higher and supports the results of this study. In this context, the results of our study emphasise the effect of haemogram parameters on PV, contrary to the results of the studies which suggest that PLT and HCT data have no effect on PV.^39^ In this context, although high PLT levels have been associated with PV in previous clinical studies, the number of studies on the effects of other haemogram parameters such as HCT, WBC and HGB on PV is limited. In this context, our research findings emphasise the importance of different blood parameters in the diagnosis of PV with AI-based modelling.

### Limitations of the study

Although the accuracy rate is high in the results of our research, our research has some limitations. One of the limitations of our research is that the data were collected from only one hospital data set. It is thought that it is important to conduct research with wider participation from different geographical regions. In addition, additional information such as symptoms, clinical findings and other biochemical data were not used in this study. This study collected the data of the participants in a retrospective manner. However, it is thought that in addition to the short-term prediction performance of the model, its long-term effect should also be examined. Another important limitation of our study may be that some of the patients with a diagnosis of haemoglobin elevation did not accept further analyses such as *JAK2* and bone marrow biopsy. In future studies, a higher level of prediction performance can be achieved by excluding these participants from the study. In addition, data on smoking, chronic obstructive pulmonary disease, obesity and sleep apnoea were not complete in our study. In future studies, these parameters can be included in the model and analyses with higher accuracy can be performed.

## Conclusion

The number of patients evaluated in advanced clinics due to erythrocytosis is quite high. Only 5.5% of these patients are detected as PV. The tests performed during this evaluation are quite costly and time-consuming since they are performed in specialised clinics. For this reason, the ML algorithm created with the help of CBC parameters in our study diagnoses PV with 94% accuracy. Therefore, it is thought that the results of our study should be utilised in the diagnosis and treatment of PV. In addition, our results can be used as a preliminary diagnostic criterion before *JAK2* and bone marrow biopsy tests. In addition, this study may lead to the generalisation of the use of ML and AI algorithms in the prediction of different diseases. Based on our current research findings, models can be created in which the effects of different external factors (smoking, high altitude, different diseases, etc) on PV are examined and physiological and environmental factors are evaluated together.

## Supplementary material

10.1136/jcp-2025-210087 online supplemental file 1

## Data Availability

Data are available upon reasonable request.

## References

[1] Mithoowani S, Laureano M, Crowther MA (2020). Investigation and management of erythrocytosis. CMAJ.

[2] Noumani I, Harrison CN, McMullin MF (2024). Erythrocytosis: Diagnosis and investigation. Int J Lab Hematol.

[3] Keohane C, McMullin MF, Harrison C (2013). The diagnosis and management of erythrocytosis. BMJ.

[4] Barbui T, Thiele J, Gisslinger H (2018). The 2016 WHO classification and diagnostic criteria for myeloproliferative neoplasms: document summary and in-depth discussion. Blood Cancer J.

[5] McMullin MF (2008). The classification and diagnosis of erythrocytosis. Int J Lab Hematol.

[6] Tefferi A, Vannucchi AM, Barbui T (2021). Polycythemia vera: historical oversights, diagnostic details, and therapeutic views. Leukemia.

[7] Andıç N, Ünübol M, Yağcı E (2016). Clinical Features of 294 Turkish Patients with Chronic Myeloproliferative Neoplasms. *Turk J Haematol*.

[8] Tefferi A, Rumi E, Finazzi G (2013). Survival and prognosis among 1545 patients with contemporary polycythemia vera: an international study. Leukemia.

[9] Landolfi R, Di Gennaro L, Barbui T (2007). Leukocytosis as a major thrombotic risk factor in patients with polycythemia vera. Blood.

[10] Skoda RC, Duek A, Grisouard J (2015). Pathogenesis of myeloproliferative neoplasms. Exp Hematol.

[11] Tefferi A, Barbui T (2023). Polycythemia vera: 2024 update on diagnosis, risk‐stratification, and management. American J Hematol.

[12] Kohli PS, Arora S (2018). Application of machine learning in disease prediction.

[13] Mujumdar A, Vaidehi V (2019). Diabetes Prediction using Machine Learning Algorithms. Procedia Comput Sci.

[14] Uddin S, Khan A, Hossain ME (2019). Comparing different supervised machine learning algorithms for disease prediction. BMC Med Inform Decis Mak.

[15] Islam R, Sultana A, Islam MR (2024). A comprehensive review for chronic disease prediction using machine learning algorithms. Journal of Electrical Systems and Inf Technol.

[16] Hamilton RI, Papadopoulos PN (2024). Using SHAP Values and Machine Learning to Understand Trends in the Transient Stability Limit. IEEE Trans Power Syst.

[17] Poodt J, Fijnheer R, Walsh IBB (2006). A sensitive and reliable semi-quantitative real-time PCR assay to detect JAK2 V617F in blood. Hematol Oncol.

[18] Orazi A (2007). Histopathology in the diagnosis and classification of acute myeloid leukemia, myelodysplastic syndromes, and myelodysplastic/myeloproliferative diseases. Pathobiology.

[19] Toll L, Weiss N, Vierbaum L (2024). Longitudinal evaluation of laboratory results and method precision in worldwide erythropoietin external quality assessments. Front Mol Biosci.

[20] Mossuz P, Girodon F, Hermouet S (2005). Serum erythropoietin measured by chemiluminescent immunometric assay: an accurate diagnostic test for absolute erythrocytosis. Clin Chem.

[21] Dong J, Feng T, Thapa-Chhetry B (2021). Machine learning model for early prediction of acute kidney injury (AKI) in pediatric critical care. Crit Care.

[22] Aromolaran O, Aromolaran D, Isewon I (2021). Machine learning approach to gene essentiality prediction: a review. *Brief Bioinform*.

[23] Gök EC, Olgun MO (2021). SMOTE-NC and gradient boosting imputation based random forest classifier for predicting severity level of covid-19 patients with blood samples. Neural Comput Appl.

[24] Gholampour S (2024). Impact of Nature of Medical Data on Machine and Deep Learning for Imbalanced Datasets: Clinical Validity of SMOTE Is Questionable. MAKE.

[25] Zhang A, Yu H, Huan Z (2022). SMOTE-RkNN: A hybrid re-sampling method based on SMOTE and reverse k-nearest neighbors. Inf Sci (Ny).

[26] Parmar A, Katariya R, Patel V (2019). A review on random forest: an ensemble classifier.

[27] Ibrahem Ahmed Osman A, Najah Ahmed A, Chow MF (2021). Extreme gradient boosting (Xgboost) model to predict the groundwater levels in Selangor Malaysia. *Ain Shams Engineering Journal*.

[28] Uddin S, Haque I, Lu H (2022). Comparative performance analysis of K-nearest neighbour (KNN) algorithm and its different variants for disease prediction. Sci Rep.

[29] Nanthini K, Pyingkodi M, Sivabalaselvamani D (2023). Performance analysis of machine learning algorithms in heart diseases prediction.

[30] Iurlo A, Cattaneo D, Bucelli C (2020). New Perspectives on Polycythemia Vera: From Diagnosis to Therapy. Int J Mol Sci.

[31] Davila-Gonzalez D, Barrios-Ruiz A, Fountain E (2021). Diagnostic Performance of Erythropoietin Levels in Polycythemia Vera: Experience at a Comprehensive Cancer Center. Clin Lymphoma Myeloma Leuk.

[32] Silver RT, Abu-Zeinah G (2023). Polycythemia vera: aspects of its current diagnosis and initial treatment. Expert Rev Hematol.

[33] Barrios-Ruiz A, Davila-Gonzalez D, Fountain E (2022). Potential limitations of diagnostic standard codes to distinguish polycythemia vera and secondary erythrocytosis. Sci Rep.

[34] Shah D, Patel S, Bharti SK (2020). Heart Disease Prediction using Machine Learning Techniques. SN COMPUT SCI.

[35] Arumugam K, Naved M, Shinde PP (2023). Multiple disease prediction using Machine learning algorithms. Mater Today.

[36] Zhang X, Yan C, Gao C (2020). Predicting Missing Values in Medical Data via XGBoost Regression. *J Healthc Inform Res*.

[37] Zhang X, Tian Z (2023). Modal Parameter Identification of Civil Engineering Structure Based on XGBoost Algorithm. Procedia Comput Sci.

[38] Krečak I, Holik H, Morić Perić M (2022). High platelet-to-lymphocyte ratio may differentiate polycythemia vera from secondary polycythemia. Wien Klin Wochenschr.

[39] Di Nisio M, Barbui T, Di Gennaro L (2007). The haematocrit and platelet target in polycythemia vera. Br J Haematol.

